# Incipient Insanity

**Published:** 1887-12

**Authors:** H. E. Stroud

**Affiliations:** Grand Junction, Col.


### INCIPIENT INSANITY.

H. E. STROUD, M. D., GRAND JUNCTION, COL.

In August 1885, Mr. M. brought his wife to me to see what could be done for her. Her symptoms were as follows:

Pains all over the body, spine and head; great tenderness of the whole length of the spine; hallucinations, spectres, dreams; during spells of excitement she would hear some one calling her and wander away from home, once walking into the river; frequently threatening suicide; often lying awake all night, crying, laughing, praying and answering imaginary voices. On such occasions she would smoke incessantly, frequently as much as a quarter of a pound of tobacco a night. Several times she had wandered away from home, and was found several miles distant. These paroxysms were followed by great prostration and deep sleep, frequently falling insensible, apparently asleep, from which she could not be aroused for usually some hours. Upon awakening, she would be almost rational for a few hours or days, at most.

She had suffered much from neuralgia, and three years before had commenced to take opium to relieve it. As they lived on a cattle ranch away from town, she could not get opium very often, and had never taken it regularly; but, during a paroxysm, had frequently taken as much as four ounces of laudanum a night.

She had been a school and music teacher; later, had kept hotel in the mining camps. She had for years studied and worked very hard. She was suspicious of every one, especially her husband ; thought he was trying to get her in an asylum. So the poor woman was nearly crazy.

I proscribed opium, tobacco, and, in fact, everything that would tend to irritate the nerve centres, and put her upon bromides, soda and potass.; also some anti-spasmodic pills to be used in case of emergency ; very light or no work, nutritious diet, gentle exercise and counter-irritants to opium. This was about the treatment (with some variation) that was kept up for about a year. I saw her from every three to eight weeks, but could notice but little improvement. Her husband, thoroughly discouraged, was seriously contemplating putting her into an asylum. I had frequently asked him to bring her to town for a few weeks, that I might try the effect of the battery ; but the lady always refused, saying that she would worry about her cows. But on August 24, 1886, she came and stayed until September 6. I commenced to use the Faradic current to the spine every day, placing one sponge on the neck and slowly drawing the other the whole length of the spine. After a few days I could use a much stronger current, and soon each treatment was finished by using the metallic brush five or six times. Three times a day she took one of DeFoe's valerianate of iron, quinine and zinc pills (aa gr. j) before meals, followed by 10 grains lactopeptine, after eating. At bedtime she took 7½ grains each, bromide soda and potass. This treatment was continued for three weeks with the most surprising results. The spine, which was so extremely sensitive, now could bear firm pressure without pain. The mind is wonderfully restored ; no hallucinations, no frenzies ; she sleeps and eats well without bromide and tonics ; she is, in fact, a changed woman.

It is now several months since she left, and I hear of her frequently. She is working hard, and has had two nervous attacks brought on by over-exertion. From the improvement that was worked in the short time, I feel convinced she would recover if it was continued, and no relapse brought on by overwork and anxiety. Just which part of the treatment deserves the most credit, I will not judge, but I have great confidence in De Foe's pills in the incipient insanity of overworked nervous women. I should state that the lady's age is fifty-two. My last report of her was that she was quite well.

